# Early vs. Late Tracheostomy in Patients with Traumatic Brain Injury: Systematic Review and Meta-Analysis

**DOI:** 10.3390/jcm10153319

**Published:** 2021-07-28

**Authors:** Annachiara Marra, Maria Vargas, Pasquale Buonanno, Carmine Iacovazzo, Antonio Coviello, Giuseppe Servillo

**Affiliations:** Department of Neurosciences, Reproductive and Odontostomatological Sciences, University of Naples “Federico II”, 80100 Naples, Italy; dottmarraannachiara@gmail.com (A.M.); pasqual3.buonanno@gmail.com (P.B.); dott.iacovazzo@gmail.com (C.I.); antonio_coviello@live.it (A.C.); maria.vargas@unina.it (G.S.)

**Keywords:** acute brain injury, early tracheostomy, late tracheostomy, tracheostomy timing, mortality, ventilatory acquired pneumonia

## Abstract

**Introduction.** Tracheostomy can help weaning in long-term ventilated patients, reducing the duration of mechanical ventilation and intensive care unit length of stay, and decreasing complications from prolonged tracheal intubation. In traumatic brain injury (TBI), ideal timing for tracheostomy is still debated. We performed a systematic review and meta-analysis to evaluate the effects of timing (early vs. late) of tracheostomy on mortality and incidence of VAP in traumatic brain-injured patients. **Methods.** This study was conducted in conformity with the Preferred Reporting Items for Systematic Reviews and Meta-Analyses (PRISMA) guideline. We performed a search in PubMed, using an association between heading terms: early, tracheostomy, TBI, prognosis, recovery, impact, mortality, morbidity, and brain trauma OR brain injury. Two reviewers independently assessed the methodological quality of eligible studies using the Newcastle–Ottawa Scale (NOS). Comparative analyses were made among Early Tracheostomy (ET) and late tracheostomy (LT) groups. Our primary outcome was the odds ratio of mortality and incidence of VAP between the ET and LT groups in acute brain injury patients. Secondary outcomes included the standardized mean difference (MD) of the duration of mechanical ventilation, ICU length of stay (LOS), and hospital LOS. **Results.** We included two randomized controlled trials, three observational trials, one cross-sectional study, and three retrospective cohort studies. The total number of participants in the ET group was 2509, while in the LT group it was 2597. Early tracheostomy reduced risk for incidence of pneumonia, ICU length of stay, hospital length of stay and duration of mechanical ventilation, but not mortality. **Conclusions.** In TBI patients, early tracheostomy compared with late tracheostomy might reduce risk for VAP, ICU and hospital LOS, and duration of mechanical ventilation, but increase the risk of mortality.

## 1. Introduction

Traumatic brain injury (TBI) is a complex disorder which can affect the central nervous system, leading to temporary or permanent physical, cognitive, and psychosocial impairments [1]. The worldwide incidence of TBI is estimated at 939 cases per 100,000 people with the highest peak of incidence in North America and Europe [2].

In patients with TBI, endotracheal intubation is often necessary to maintain airway patency and prevent hypoxia [3]. Tracheostomy may facilitate weaning in long-term mechanical ventilated patients, reduce duration of intensive care unit (ICU) length of stay (LOS), and decrease complications from prolonged tracheal intubation [4,5].

In TBI patients, the main indications for tracheostomy include weaning failure, absence of protective airway reflexes, impairment of respiratory drive, and difficulties in managing secretions [6]. However, the beneficial effects, timing and indications of tracheostomy in TBI are still debating [7,8].

In ICU patients, the use of tracheostomy may improve the comfort of patients, allow more effective secretions suctioning and a more secure airway, decrease airway resistance, enhance patient mobility, opportunities for speech and eating orally. Early and late complications after tracheotomy include bleeding, wound infection, subcutaneous emphysema, laryngeal nerve or esophageal injury, and tracheal stenosis.

Tracheostomies performed during the first week of mechanical ventilation are classified as early, while tracheostomies performed later than seven days are defined as late [9]. Evidence on the advantages of early over late tracheostomy is conflicting [5], and there are limited robust data to guide the ideal timing to perform a tracheostomy.

Systematic reviews of randomized controlled trials (RCTs) in general critical care populations have generally not found benefit from early tracheostomy [8,9,10], but these results cannot be generalized to traumatic brain-injured patients, who typically require tracheostomy for airway protection for depressed airway reflexes rather than respiratory failure.

Observational studies in traumatic brain-injured patients suggest that tracheostomy performed earlier may be associated with lower in-hospital morbidity and improved clinical outcomes [11,12,13,14], but the best timing for tracheostomy continues to be debated.

To address these gaps in knowledge, we performed a systematic review and meta-analysis to evaluate the effects of early vs. late tracheostomy on mortality and VAP incidence in acutely brain-injured patients.

## 2. Materials and Methods

Our study was conducted according to the Preferred Reporting Items for Systematic Reviews and Meta-Analyses (PRISMA) guideline [15]. The following terms were used to perform a PubMed search: early, tracheostomy, TBI, prognosis, recovery, impact, mortality, morbidity, and brain trauma OR brain injury. Inclusion criteria were (1) English language; (2) TBI as the main cause of trauma; (3) clear outcome; (4) reliable patient’s admission assessment; (5) late tracheostomy (LT) clearly defined and not confused with prolonged intubation; and (6) a minimum of two outcomes: ICU stay, hospital stay, mortality rates, or ventilator-associated pneumonia (VAP) diagnosis. We included randomized controlled studies, retrospective and prospective studies. We excluded studies without full reports or abstracts, commentaries, editorials, and reviews.

## 3. Data Extraction

Two reviewers (A.M. and M.V.) independently screened studies for inclusion, retrieved potentially relevant studies, and decided on study eligibility using a standardized data extraction form, checked by the other authors. Any disagreement was solved by discussion or by the judgment of a third author (P.B.). We collected the following data from every study included in our analysis: study design, year, patient’s demographics, mean time between admission and tracheostomy, neurologic assessment at admission, confirmed VAP, median ICU stay, median hospital stay, mortality rates, and ICU or hospital costs. Two investigators (P.B. and C.I.) independently screened the citations to identify other potentially eligible studies not included in the previous PubMed search.

## 4. Risk of Bias

Two reviewers (M.V. and P.B.) independently assessed the methodological quality of eligible studies using the Newcastle–Ottawa Scale (NOS) for assessing the quality of nonrandomized studies in a meta-analysis [16] for each included trial. Any disagreement was resolved asking for the opinion of a third reviewer (G.S.).

## 5. Data Synthesis and Analysis

The primary outcome were the odds ratio of mortality and the incidence of VAP between the early tracheostomy (ET) and LT groups. The secondary outcomes were the duration of mechanical ventilation, ICU length of stay (LOS), and hospital LOS. A standardized mean difference was used as effect size to compare the two groups. Consequently, random effects model was used [17]. This model is more conservative and reduces the likelihood of type II errors. Heterogeneity was assessed by I^2^ calculation, and it was considered low, moderate, or high if I^2^ values were 25%, 50%, and 75%, respectively. Results expressed with median and range were converted in mean and standard deviation according to Hozo et al. [18]. Trial sequential analysis (TSA) was performed to determine the required information size (RIS), i.e., the number of subjects to enroll in order to confirm or reject the supposed effect of an intervention. TSA was undertaken using TSA 0.9 beta software if the number of included trials was more than five. The RIS was estimated using relative risk reduction and heterogeneity-adjusted information size for dichotomous outcomes. Results are considered conclusive if the cumulative Z-curve crosses the conventional significance boundary (Z = 1.96) or the trial sequential boundary (i.e., significance or futility boundaries) or if the RIS is reached. TSA-adjusted 95% CIs were also presented.

## 6. Results

**Figure 1:**
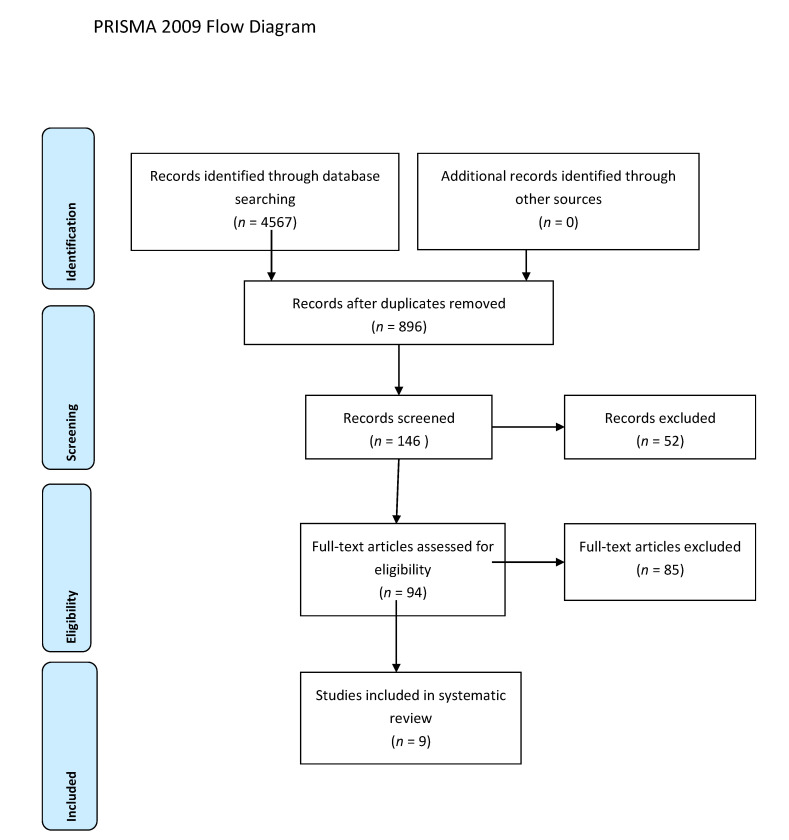
PRISMA 2009 Flow Diagram.

**Figure 2:**
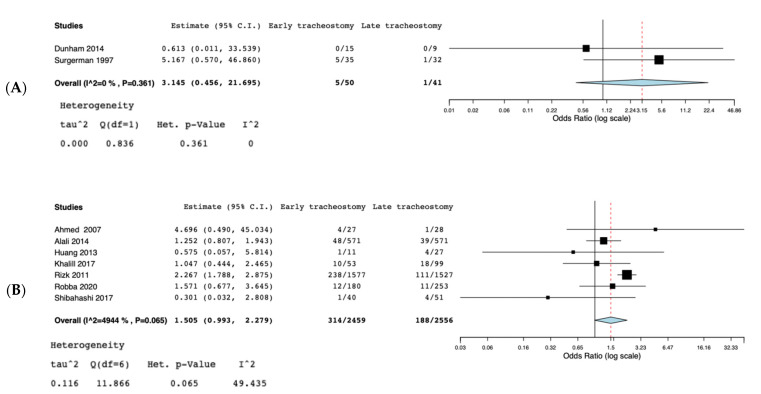
(**A**): Forest plot for incidence of pneumonia; (**B**): Forest plot for incidence of pneumonia in RCTs.

**Figure 3:**
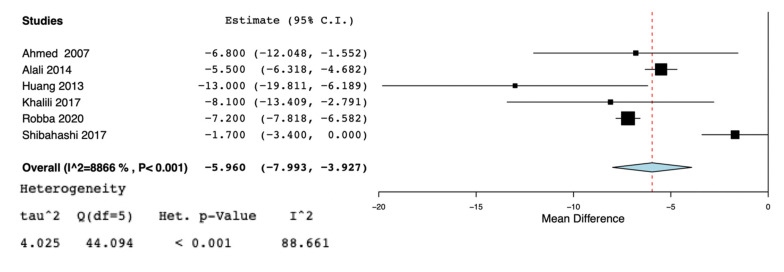
Forest plot for ICU length of stay.

**Figure 4:**
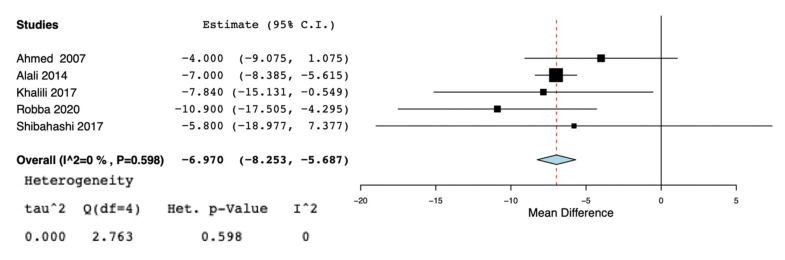
Forest plot for hospital length of stay.

**Figure 5:**
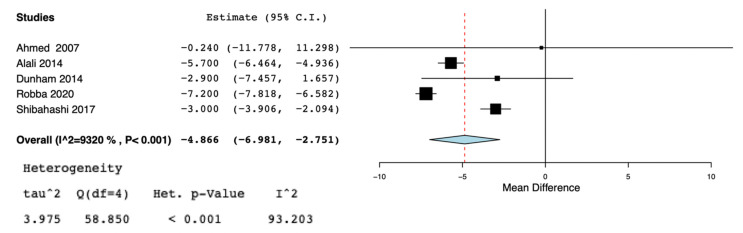
Forest plot for duration of mechanical ventilation.

**Figure 6:**
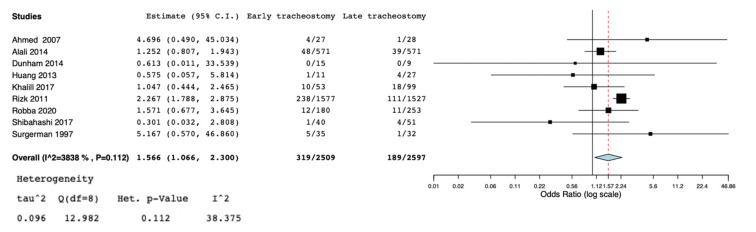
Forest plot for mortality in included studies.

A total of nine studies [5,11,13,19,20,21,22,23,24] were selected for the systematic review (Figure 1) (Table 1). According to the NOS [16], the quality scores of the included studies ranged from 5 to 8. Most of them (7/9) were greater than or equal to seven stars, as listed. We included two randomized controlled trials [19,24], three observational trials [5,13,21], one cross-sectional study [20], and three retrospective cohort studies [11,22,23]. Great heterogeneity was observed in the definition of the early tracheostomy. Shibahashi et al. [22] performed tracheostomy within 72 h after admission, in two studies [19,24] early tracheostomy was performed on post-injury day 3–5, while, in Khalili et al. [21], ET was performed before or at the sixth day of admission, in 2 other studies [5,23] early tracheostomy was performed ≤7 days from admission, Alali et al. [11] classified as early tracheostomy a procedure executed ≤8 days, and in 2 other studies [13,20] ET was defined as the performance of the procedure within the first 10 days of mechanical ventilation or after decompressive craniectomy. The total number of participants in the ET group was 2509, while in the LT group it was 2597. Reduced risk for incidence of pneumonia was found in the ET group (OR = 0.63, 95% CI = 0.52, 0.76, I^2^ = 0%, *p* = 0.89) (Figure 2), but this result was confirmed only by the analysis including the prospective and retrospective studies but not the RCTs (OR = 0.62, 95% CI = 0.51, 0.75, I^2^ = 0%, *p* = 0.71) (Supplemental Figure S1). ET was significantly associated to reduced ICU length of stay (MD = −5.96, 95% CI = −7,99, −3.92, I^2^ = 88%, *p* < 0.001) (Figure 3), hospital length of stay (MD = −6.97, 95% CI= −8.25, −5.68, I^2^ = 0%, *p* = 0.59) (Figure 4), and duration of mechanical ventilation (MD = −4.86.56, 95% CI= −6.98, −2.75, I^2^ = 93%, *p* < 0.001) (Figure 5). Increased risk of mortality was found in the ET group (OR = 1.56, 95%CI = 1.06, 2.3, I^2^ = 38.3%, *p* = 0.11) (Figure 6; Supplemental Figure S2). The TSA adjusted 95% CI was ranged from 0.57 to 46.86. The cumulative z-curve crossed neither the conventional boundary for benefit nor the trial sequential futility boundary for benefit, suggesting that the current evidence was inconclusive (Supplemental Figure S3). Furthermore, we need 151 from randomized controlled trials to assess the impact of ET on mortality.

## 7. Discussion

In this systematic review involving 9 studies and 5106 patients, we found that early tracheostomy, compared with late tracheostomy, might reduce risk for VAP, ICU and hospital LOS, and duration of mechanical ventilation, while an increased risk of mortality was found in the LT group.

Tracheostomy is a common procedure performed in critically ill patients. Patients with severe TBI may need prolonged MV to avoid complications such as hypoxemia and hypercapnia [13]. Robba et al. [5] found that TBI patients underwent tracheostomy more frequently than the general ICU population (31.8% vs. 10%, respectively) [25,26]; this could be due to a higher risk of extubation failure, and the impairment of airways protection reflexes secondary to the neurological injury.

In ICU patients, tracheostomy is most commonly performed after 14 days from admission [27,28], and only a quarter of tracheostomies are accomplished in the first week [25]. In TBI patients, multiple factors, related to severity of neurological injury, pre- and post-hospitalization management, evolution of trauma, local medical practices, ethical and legal implications, and costs [5,25,29,30], play a role in the decision-making process of whether and when to perform the tracheotomy. Literature reported a median time to tracheostomy of 9 days post-admission, probably reflecting a change in treatment goals [5] no longer aimed to manage acute intracranial emergencies, but focused on weaning from ventilator support and rehabilitation [5]. Moreover, this timing of tracheostomy also prevents the use of the procedure in patients with lesser or higher severities of injury; in the former case, patients have enough time to recover spontaneous breathing and an adequate level of consciousness, in the latter case they succumb early because of the rapid progression of the lesions [5].

This process still leads to performing tracheostomy at an earlier stage than in patients without TBI, but allows for the identification of patients who are most likely to benefit from the procedure [21,31,32,33,34,35].

Pneumonia, especially VAP, is one of the major complications of TBI that adversely affects outcome, and its risk showed a 10% increase per day of mechanical ventilation [36,37]. Similarly to De Franca et al., our results show that ET, compared with LT, might reduce the risk for VAP, probably due to the reduction of ventilation days of ET compared to LT [1,3,8,38].

Like other literature reports, we found that early tracheostomy may potentially reduce hospital stay, duration of mechanical ventilation and mortality rates [1,3,23,24,31,39]. In a propensity-matched cohort study on TBI patients, early tracheostomy (≤7 days) was associated with shorter ICU and hospital LOS but did not affect mortality [11]. Khalili et al. [21], in a cohort of 152 TBI patients, showed similar results on ICU and hospital LOS and mortality. A meta-analysis by McCredie et al. [39] found that ET might reduce the long-term mortality, duration of mechanical ventilation, and LOS. Robba et al. [5] found that each delay of 1 day to perform a tracheostomy was associated with a 4% increase in the risk of an unfavorable outcome and with a 6% increase in the hazard of death. While this association may suggest a benefit from an ET, patients with more severe injury may need more time to control the intracranial damage evolution and stabilize their condition, thus delaying tracheostomy, or may have a worse expected outcome, restraining the decision for the tracheostomy. The same study showed that patients who received LT had a significant longer ICU and hospital LOS; for every 2 days of deferral in tracheostomy, ICU and hospital LOS increase of about 1 and 2 days, respectively [5]. De Franca et al. [1] demonstrated that patients undergoing ET had a shorter ICU and hospital stay, which can reflect the impact of tracheostomy in patient recovery from hemodynamic instability and in a faster weaning from mechanical ventilation.

We found that the LT group had an increased risk of mortality. Conversely, Lu et al. [3], as in other previous finding [8,40,41,42,43], found no differences in mortality between the ET and not-ET groups. These studies showed improved outcomes for the ET groups with no survival benefit. The high mortality rate in tracheostomy patients could be related to the complications of tracheostomy (e.g., wound infection, esophageal injury, pneumothorax, and tracheal stenosis) [44]; moreover, the majority of patients undergoing tracheostomy are in severe clinical conditions with a probable high risk of death [45], which can influence the statistical significance of mortality rates. Our results could depend on the fact that mortality in ICU is a complex outcome, taking into account different variables including age, sex, comorbidities, and the length of follow-up time. According to this, mortality could be not driven by a single parameter like timing of tracheostomy, that is, a procedure that may allow a better management of critically ill patients. In addition, results of TSA suggested that current evidence is inconclusive and that more randomized controlled trials are necessary to assess the real impact of ET on mortality.

Despite the known advantages, there are still some controversies regarding tracheotomy in TBI. According to Cox et al., tracheostomy increases the proportion of patients with chronic burden, contributing to raising the costs outside the hospital [44,46].

This systematic review and meta-analysis added several novelties compared to the current literature. We included the huge study of Robba et al. [5] that selected TBI patients from CENTER-TBI, a prospective observational longitudinal cohort study, including 1358 patients, of which 433 (31.8%) had a tracheostomy. Moreover, in our systematic review and meta-analysis, we carried out a sub-group analysis according to the type of the included studies (RCT vs. non RCT) and performed a trial sequential analysis on RCTs.

Of note, this meta-analysis has some limitations. First, there is the ambiguous definition of timing to differentiate an early and a late tracheostomy. Second, heterogeneity more than 50% was found in 2 out of 5 considered outcomes like ICU LOS and duration of mechanical ventilation. Third, only published articles were reviewed, which might have contributed to a publication bias.

## 8. Conclusions and Future Perspectives

Our meta-analysis suggests that ET in TBI patients could help in reducing duration of mechanical ventilation, ICU and hospital LOS, contribute to a lower exposure to secondary injuries and nosocomial adverse events, increasing the opportunity of patients’ early rehabilitation and discharge.

Further studies, especially multicenter RCTs, are needed to collect more data about the different outcomes of TBI patients undergoing ET compared to those treated with LT in order to confirm the superiority of the former airway management in such a challenging clinical condition.

## Figures and Tables

**Table 1:** Characteristics of the studies included in this meta-analysis. NA = not available.

Authors	Study Design	Age (Years) in ET vs. LT Groups	Sex (Male) in ET vs. LT Groups	GCS Score Information ET vs. LT Groups	How Early Tracheostomy Was Defined by the Studies
Alali et al. [12]	Retrospective cohort	49 (30–64) vs. 53 (35–68)	75.6% vs. 73%	4 (3–7) vs. 7 (3–13)	≤8 days
Bouderka et al. [23]	Retrospective cohort	NA	NA	NA	<7 days
Dunham et al. [19]	Randomized controlled trial	NA	NA	NA	3–5 days of endotracheal tube
Huang et al. [20]	Cross-sectional study	NA	NA	NA	≤10 days after decompressive tracheotomy
Khalili et al. [21]	Observational cohort	41.6 vs. 37.8	50% vs. 86%	6.15 vs. 5.70	≤6 days
Robba et al. [6]	Prospective observational	48.5 (31–67) vs. 44 (28–59	77.2% vs. 76.7%	5.5 (3–10 vs. 5 (3–9)	≤7 days
Shibahashi et al. [22]	Retrospective cohort	68 (62–74) vs. 68 (53–74)	33 vs. 33	6 (3–7) vs. 6 (6–9)	≤3 days
Surgeman et al. [24]	Randomized controlled trial	NA	NA	NA	3–5 days
Wang et al. [14]	Observational cohort	55.3 (19–80) vs. 57.5 (18–85)	87.5% vs. 66%	5.9 (3–8) vs. 5.7 83–8)	≤10 days

## Data Availability

Not applicable.

## Supplementary Materials

The following are available online at https://www.mdpi.com/article/10.3390/jcm10153319/s1, Figure S1: Forest plot for incidence of pneumonia in non RCTs, Figure S2: A: Forest plot for mortality in RCTs; B: forest plot for mortality in non RCTs, Figure S3: Trial Sequential Analysis on mortality.
