# Supplementary material for: Early vs. Late Tracheostomy in Patients with Traumatic Brain Injury: Systematic Review and Meta-Analysis
Source: J Clin Med. 2021 Jul 28;10(15):3319. doi: 10.3390/jcm10153319 (PMC8348593; doi:10.3390/jcm10153319)

Supplemental Figure S1: Forest plot for incidence of pneumonia in non RCTs

| Studies | Estimate (95% C.I.) | Early tracheostomy | Late tracheostomy |
|---|---|---|---|
| Ahmed 2007 | 0.688 (0.237, 1.998) | 11/27 | 14/28 |
| Alali 2014 | 0.641 (0.507, 0.810) | 238/571 | 301/571 |
| Khalill 2017 | 0.759 (0.388, 1.487) | 28/53 | 59/99 |
| Robba 2020 | 0.572 (0.378, 0.866) | 49/180 | 100/253 |
| Shibahashi 2017 | 0.688 (0.290, 1.634) | 13/40 | 21/51 |
| Wang 2012 | 0.246 (0.075, 0.801) | 7/16 | 38/50 |
| **Overall ($I^2$=0 % , P=0.697)** | **0.623 (0.518, 0.750)** | **346/887** | **533/1052** |

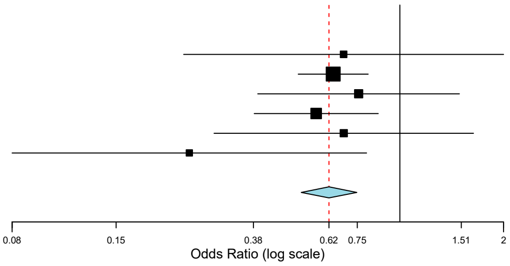

Supplementary Figure S2: A: Forest plot for mortality in RCTs; B: forest plot for mortality in non RCTs.

| Studies | Estimate (95% C.I.) | Early tracheostomy | Late tracheostomy |
|---|---|---|---|
| Dunham 2014 | 0.613 (0.011, 33.539) | 0/15 | 0/9 |
| Surgerman 1997 | 5.167 (0.570, 46.860) | 5/35 | 1/32 |
| **Overall (I^2=0 % , P=0.361)** | **3.145 (0.456, 21.695)** | **5/50** | **1/41** |

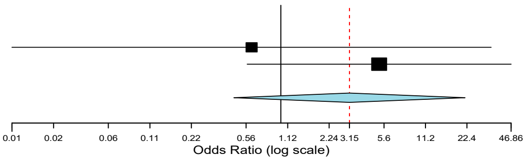


**Heterogeneity**

| tau^2 | Q(df=1) | Het. p-Value | I^2 |
|---|---|---|---|
| 0.000 | 0.836 | 0.361 | 0 |

| Studies | Estimate (95% C.I.) | Early tracheostomy | Late tracheostomy |
|---|---|---|---|
| Ahmed 2007 | 4.696 (0.490, 45.034) | 4/27 | 1/28 |
| Alali 2014 | 1.252 (0.807, 1.943) | 48/571 | 39/571 |
| Huang 2013 | 0.575 (0.057, 5.814) | 1/11 | 4/27 |
| Khalill 2017 | 1.047 (0.444, 2.465) | 10/53 | 18/99 |
| Rizk 2011 | 2.267 (1.788, 2.875) | 238/1577 | 111/1527 |
| Robba 2020 | 1.571 (0.677, 3.645) | 12/180 | 11/253 |
| Shibahashi 2017 | 0.301 (0.032, 2.808) | 1/40 | 4/51 |
| **Overall (I^2=4944 % , P=0.065)** | **1.505 (0.993, 2.279)** | **314/2459** | **188/2556** |

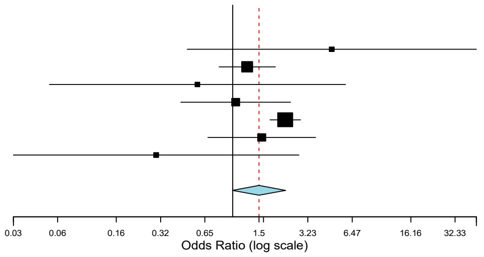


**Heterogeneity**

| tau^2 | Q(df=6) | Het. p-Value | I^2 |
|---|---|---|---|
| 0.116 | 11.866 | 0.065 | 49.435 |

Supplemental Figure S3: Trial Sequential Analysis on mortality.

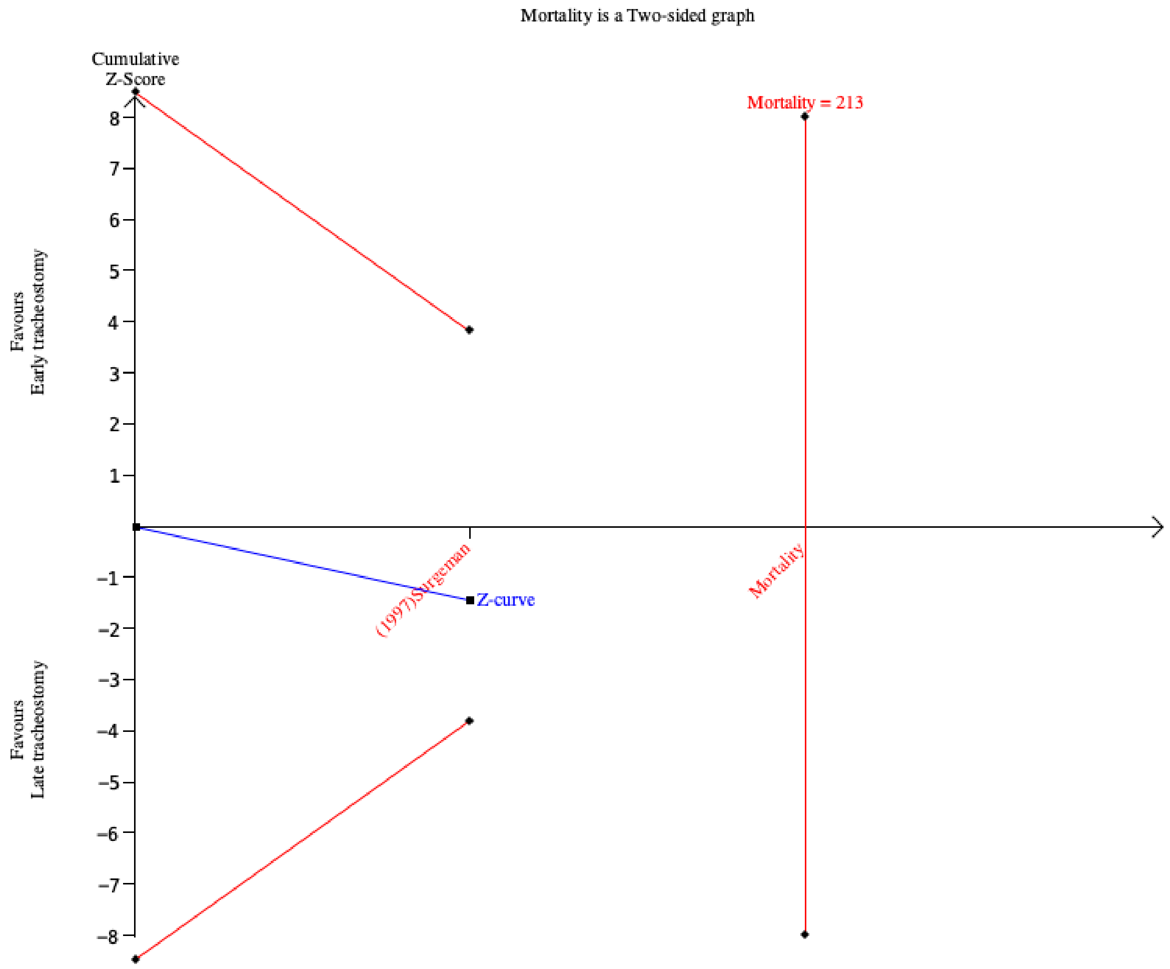

Supplement: Supplementary file 1 [file jcm-10-03319-s001.zip › jcm-1305912-supplementary.pdf]
